# Excretion of enterovirus 71 in persons infected with hand, foot and mouth disease

**DOI:** 10.1186/1743-422X-10-31

**Published:** 2013-01-23

**Authors:** Jie Li, Changying Lin, Mei Qu, Xinyu Li, Zhiyong Gao, Xin Zhang, Yuan Liu, Ying Huang, Xiaoli Wang, Lei Jia, Xitai Li, Guirong Liu, Hanqiu Yan, Lijuan Chen, Quanyi Wang

**Affiliations:** 1 Institute for Infectious Disease and Endemic Disease Control, Beijing Center for Disease Prevention and Control, No.16, Hepingli middle Road, Beijing, 100013, People’s Republic of China

**Keywords:** Hand, foot and mouth disease, Enterovirus 71, Virus excretion

## Abstract

**Background:**

Hand, foot and mouth disease (HFMD) is a common illness in young children. It also can be seen in adults occasionally. Enterovirus 71 (EV71), a pathogen that causes not only HFMD but also neurological complications and even death, has caused many HFMD outbreaks in China. However, till now the data about the duration of EV71 shedding is very limited.

**Results:**

A total of 136 throat swabs and fecal samples were collected from 27 children and 3 adults, which includs 7 close contacts, 9 mild cases and 14 severe cases,. The participants were divided into three groups namely, severe case group, mild case group and close contact group. All the samples were assayed with real-time polymerase chain reaction (PCR). Kruskal-Wallis Test was employed to compare the difference in duration of viral RNA shedding among three groups. The results showed that significant difference in duration of EV71 shedding was found among three groups (P < 0.01). The longest duration of EV71 shedding in fecal samples is 54 days and 30 days in throat swabs.

**Conclusions:**

HFMD is characterized by extended excretion of EV71. Our results suggest that the duration of EV71 shedding is correlated with the severity of the disease. EV71 shedding through feces can persist more than 54 days. Prolonged virus shedding is a potential risk factor of proliferating HFMD epidemic.

## Background

Hand, Foot and Mouth disease (HFMD) is a common infectious illness. It is primarily a disease among children but occasionally, it can be seen in immunocompromised or immunocompetent adults [1-3]. This disease can be found all over the world. A number of enteroviruses can cause this disease. Enterovirus 71 (EV71) and Coxsackievirus A16 (CoxA16) are the two major pathogens for HFMD [4,5]. Most severe cases are associated with EV71 [6-8]. Many sporadic cases and outbreaks of this disease caused by EV71 have been reported [9-12]. With the increasing severity and frequency of the outbreak such as Taiwan in 1998 [11,13], Shandong province of China in 2007 [14], HFMD outbreaks have increasingly become the focus of public health issues. In order to prevent and control this disease, HFMD surveillance was brought into the observation system of contagious disease of Beijing since 2007. A series of measures have been implemented to prevent and control the spread of this disease. Since the disease is infectious and can induce severe neurological complications even death, the duration of the EV71 shedding in throats or feces of HFMD patients may have extensive implications on designing effective protective measures. Prolonged excretion of EV71 may extend the contagious period and raise the likelihood of secondary infections.

The aim of this study is to determine the duration of EV71 excretion in the throats and feces of HFMD patients and obtain more information of whether the duration of EV71 shedding was correlated with the factors of the case.

## Results

A total of 30 cases which includes 27 children and 3 adults aged from 1 to 40-year-old were enrolled in this study and were categorized into 3 groups, i.e., close contact group, mild case group, severe case group. All the specimens collected from cases and the close contacts were analyzed with real-time RT-PCR for three times and the result was the same. All the participants were EV71 positive by real-time RT-PCR. The close contacts, mild cases and the severe cases had the duration of shedding longer than 6 days, 30 days and 54 days, respectively. The severe case that shedding virus for at least 54 days failed showing up in the follow-up study for 27 days. On the 81st day the fecal sample from him was identified and the result was negative. The only one adult severe case excreted EV71 for at least 40 days. The close contact group, mild case group and severe case group turned EV71 negative with the median shedding duration of 2 days, 18 days and 25 days respectively.

Kruskal-Wallis Test was employed to compare the difference in duration of viral RNA shedding among three groups. The results showed that significant difference in duration of EV71 shedding was found among three groups (P < 0.01) (Table 1).

**Table 1:** Kruskal-Wallis Test of the difference in duration of viral RNA shedding among three groups

**Grouping variable**	**No (%) of patients**	**Mean rank**	**Chi-square**	**P value**
Close contact	7 (23.33%)	4.50	17.89	P < 0.01
Mild case	9 (30.00%)	14.89		
Severe case	14 (46.67%)	21.39		
Total	30	/	/	/

**Figure 1:**
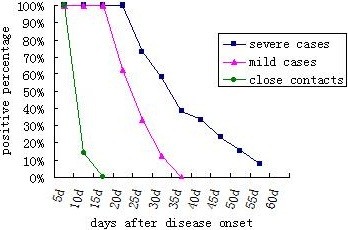
**The comparison of EV71 excretion among three paediatric groups (close contact group, mild case group, severe case group).** Solid square represents positive rates of the samples from severe case group at a certain day. Solid triangle represents positive rates of the samples from mild case group at a certain day. Solid circle represents positive rates of the samples from close contact group at a certain day.

In order to analyze the data of EV71 excretion of all the paediatric cases, the EV71 excretion of 3 groups was compared. The participants were defined as EV71 positive if the throat swab or the fecal sample was identified as EV71 positive. As showed in Figure 1, 100% severe cases were EV71 positive during the first 15 days after the onset of the disease. The positive rate decreased gradually from the 20th days. EV71 of 61.54% severe cases turned negative during the 15–35 day. The positive rate dropped to 7.69% in the 54th day after onset of the disease. All the mild cases were EV71 positive in the first week, then positive rate declined dramatically, and 87.5% mild cases turned negative during the 10-30 days. Results of close contacts were also analyzed, and they did not excrete EV71 for a long period. All the close contacts were EV71 positive at the beginning of the epidemic. The result showed that 85.7% close contacts were detected EV71 positive only once and all the close contacts turned negative in the first two weeks after the outbreak of the epidemic.

EV71 shedding of fecal samples and throat swabs from adult participants was also analyzed (Table 2). All of the specimens collected from adult close contacts turned negative in the first two weeks of the epidemic. The only one adult severe case excreted EV71 for at least 40 days.

**Table 2:** Information of samples collected from the adult participants and the corresponding testing result

**Adult group**	**The day of sample collected (D)/testing result (N/P)**						
	1w	2w	3w	4w	5w	6w	7w
Close contact 1	P	N	/	/	/	/	/
Close contact 2	P	N	/	/	/	/	/
Severe case	P	P	P	P	P	P	N

Results of fecal samples and swab samples showed that EV71 was excreted intermittently in severe cases and mild cases. 66.7% severe cases and 50% mild cases showed the phenomena of excreting EV71 intermittently both in the throat swabs and in the feces samples. A positive correlation was not found between the fecal samples and throat swabs. Fecal samples and throat samples from 2 close contacts were collected. Two days later, both of them showed the symptom of HFMD. Based on their symptoms, they are classified as mild case. Throat samples from both them were EV71 positive. Fecal sample from one case showed EV71 positive.

## Discussion

It was reported that most children experiencing infection of EV71 will cease shedding virus particles within 13 to 24 days after the disease onset [15]. The longest EV71-positive time in throat swab was reported 24 days and in feces 42 days after onset [15]. However no one reported how long the severe cases, mild cases, close contacts shed virus. This study examined the duration of EV71 excretion in sequential fecal specimens and throat swabs from mild cases, severe cases and close contacts which were definitely EV71 positive.

Using the data of this study and the Kruskal-Wallis Test, the results showed that significant difference of the duration of EV71 shedding was found among severe cases, mild cases and close contacts. This phenomenon is different from some other virus such as HAV, rotavirus, the excretion of which does not show any correlation with the severity of the disease [16,17]. Whether or not the duration of HAV excretion is correlated with the age of the patients has been reported previously [16,18]. However, the obtained results were different. Since the sample size was small, our data did not provide the information of whether the factors of age and sex influence the duration of the virus shedding. The reasons why the EV71 shedding was different among severe cases, mild cases and close contacts remain to be elucidated.

This study showed that the longest duration of EV71 shedding in fecal sample is 54 days and 30 days in throat swabs. Some researchers have reported that enterovirus shedding in fecal samples lasts for a longer period than in throat swabs [15,19,20]. EV71 is transmitted predominantly via the faeco-oral route. Prolonged excretion of EV71 will act as an on-going infection source which will play an important role in outbreaks of HFMD. This study showed that some individuals shed other enterovirus in the fecal samples after EV71 could not be detected. In this study we also found that EV71, just like rotavirus [17] or poliovirus [21], has a phenomenon of excreting virus intermittently both in fecal samples and in throat swabs after apparent recovery from initial infection. Children or adults excreting EV71 intermittently for a long time may serve as reservoirs of the virus and thus contribute to the epidemic of the HFMD.

The findings in our study suggest that patients may excrete EV71 for a long period. It means that the majority of severe cases may still be infectious during their recovery even though they are asymptomatic. The role of these prolonged excretion in perpetuating epidemics of HFMD is uncertain. In order to thoroughly eliminate the potential infective sources from the community, one solution might be for cases to be separated from other susceptible children until patients no longer excreting virus, especially for severe cases. In this study we also found that some cases were EV71 positive before they showed the typical HFMD symptoms. This phenomenon implied that cases began to excrete EV71 just at the latent period. It was a blind spot for disease prevention. So we suggest that it is necessary to make preventive measures such as sanitation, disinfection and hand-washing, especially among people at risk such as children under the age of five years old. Timely preventive measures will limit secondary cases of HFMD.

## Methods

### Case definition

Mild HFMD case definition: Mild case was defined as oral ulcers, maculopapular or vesicular rash on the hands, feet and buttock, accompanied with or without fever. Severe EV71 infection case was defined as a patient with or without the symptoms of mild case of HFMD, who had one or more symptoms which include high fever, myoclonus, encephalitis, acute flaccid paralysis, pulmonary edema, or heart failure. Close contact definition: people who spent a lot of time living with those confirmed HFMD cases.

### Patient selection and specimen collection

The epidemiological surveys of all the severe cases and their close contacts were carried out by district centers for Disease Control and Prevention. Those who were interested in this study were invited to take part in our study. Mild cases that went to see a doctor were reported on the China information system for disease control and prevention. Enrolled cases were randomly selected from those reported cases. Specimens of mild cases were collected with patient and where appropriate parental consent. A total of 136 specimens including 50 throat swabs and 86 fecal samples were collected from 30 people(close contacts, mild cases, severe cases)(Table 3). Consecutive throat swabs and fecal samples were collected from 27 children and 3 adults with laboratory confirmed EV71 infection. Samples were collected every week or every two weeks. If a patient had two consecutive EV71 PCR negative samples, no further samples were collected from them. Throat swabs were stored in a suspension of minimum essential medium (MEM). Fecal samples were stored in plastic containers. Obtained samples were stored at −70°C until analyzed.

**Table 3:** Basic information of the participants and the number of the collected samples

**Participator**	**Gender(F/M)**	**Age(year)**	**Age median**	**Throat swabs**	**Fecal samples**
**Paediatric group**					
Close contact	1/4	2~5	3	5	11
Mild case	1/8	1~5	4	10	26
Severe case	5/8	1~5	3	30	37
**Adult group**					
Close contact	1/1	37~40	/	5	6
Severe case	1/0	31	/	0	6

### Samples processing mode and RNA extraction

Just before RNA extraction, 2 g feces samples were treated with 10 ml phosphate buffer saline (PBS), 1 ml chloroform and 1 g glass bead. The mixture was vibrated thoroughly for 20 min and then centrifuged for 20 min at 1500 g. The throat swabs stored in minimum essential medium (MEM) were vibrated violently and centrifuged at 4000 g for 20 min prior to RNA extraction. RNA extraction was carried out with a Roche MagNA Pure LC 2.0 nucleic extraction system (ROCHE, Co, USE) using MagNA Pure LC Total Nucleic Acid Isolation Kit – Large Volume (ROCHE, Co, USA), according to the manufacturer’s instructions.

### Real-time RT-PCR

All the samples were analyzed with real-time RT-PCR for three times. Real-time RT-PCR was carried out with a pan-enterovirus detection kit, a EV71 detection kit and a CoxA16 detection kit(DAAN Gene, Co, CHN)according to the manufacturer’s instructions. Positive and negative controls were included in this experiment. The PCR reactions were carried out in a 96-well, 0.2 ml PCR reaction plate using the Roche 480 Real-time PCR thermocycler (ROCHE, USA) and the results were interpreted according to the manufacturer’s instructions.

#### Statistical analysis

Kruskal-Wallis Test was employed to compare the difference in duration of viral RNA shedding among three groups.

#### Ethical approval

This study was in compliance with the Helsinki Declaration and was approved by the ethics committee at Beijing center for disease control and prevention. Sample collection in this study was agreed by either the patient or the patient’s parents or guardian as appropriate with prior informed consent.

## Abbreviations

HFMD: Hand, foot and mouth disease; EV71: Enterovirus 71; CoxA16: Coxsackievirus A16; MEM: Minimum essential medium; PBS: Phosphate buffer saline; PCR: Polymerase chain reaction.

## Competing interests

The authors declare that they do not have any competing interests.

## Authors’ contributions

JL contributed to the experimental design and carried out the experiment and wrote the manuscript. CL and MQ contributed to the experimental design and participated in the experiment and reviewed the manuscript. XL and ZG contributed to the experimental design and clinical data analysis and reviewed the manuscript. XZ, YL and YH participated in the experiment and reviewed the manuscript. XW, LJ, XL, GL, HY and LC contributed to the clinical data analysis and reviewed the manuscript. QW contributed to the experimental design and coordination with District Centers for Disease Control and Prevention and provided a final review of this manuscript. All authors read and approved the final manuscript.

## References

[B1] Toya M Endo Y Tanizaki H Fujisawa A Tanioka M Miyachi Y Letter: an adult case of severe hand-foot-mouth disease accompanying persistent fever and systemic arthritis Dermatol Online J 2012 18 8 14 22948064

[B2] Shikuma E Fujisawa A Tanioka M Matsumura Y Miyachi Y Letter: an adult case of hand-foot-mouth disease showing severe mucous involvement Dermatol Online J 2011 17 12 15 22233751

[B3] Shin JU Oh SH Lee JH A case of hand-foot-mouth disease in an immunocompetent adult Ann Dermatol 2010 22 2 216 218 10.5021/ad.2010.22.2.216 20548919 PMC2883431

[B4] McMinn PC An overview of the evolution of enterovirus 71 and its clinical and public health significance FEMS Microbiol Rev 2002 26 91 107 10.1111/j.1574-6976.2002.tb00601.x 12007645

[B5] Pallansch MA Roos RP Knipe DM, Howley PM Enteroviruses: polioviruses, coxsackieviruses, echoviruses and newer enteroviruses Fields virology 2001 Volume 1. 4th Philadelphia: Lippincott Williams& Wilkins 723 775

[B6] Ishimaru Y Nakano S Yamaoka K Takami S Outbreaks of hand, foot and mouth disease by enterovirus 71: high incidence of complication disorders of central nervous system Arch Dis Child 1980 55 8 583 588 10.1136/adc.55.8.583 6254449 PMC1627055

[B7] Alexander JP Jr Baden L Pallansch MA Anderson LJ Enterovirus 71 infections and neurologic disease-UnitedStates, 1977–1991 J Infect Dis 1994 169 4 905 908 10.1093/infdis/169.4.905 8133108

[B8] Chang LY Huang YC Lin TY Fulminant neurogenic pulmonary oedema with hand, foot and mouth disease Lancet 1998 352 9125 367 368 10.1016/S0140-6736(98)24031-1 9717926

[B9] Cardosa MJ Krishnan S Tio PH Perera D Wong SC Isolation of subgenus B adenovirus during a fatal outbreak of enterovirus 71 associated hand, foot, and mouth disease in Sibu, Sarawak Lancet 1999 18 354 (9183): 987–991 10.1016/S0140-6736(98)11032-2 10501361

[B10] Gilbert GL Dickson KE Waters MJ Kennett ML Land SA Sneddon M Outbreak of enterovirus 71 in Victoria, Australia, with a high incidence of neurologic involvement Pediatr Infect Dis J 1988 7 7 484 488 10.1097/00006454-198807000-00007 2841639

[B11] Ho M Chen ER Hsu KH Twu SJ Chen KT Tsai SF Wang JR Shih SR An epidemic of enterovirus 71 infection in Taiwan. Taiwan enterovirus epidemic working group New Engl J Med 1999 23 341 (13):929–935 10.1056/NEJM199909233411301 10498487

[B12] Chan KP Goh KT Chong CY Teo ES Lau G Ling AE Epidemic hand, foot, and mouth disease caused by human enterovirus 71, Singapore Emerg Infect Dis 2003 9 1 78 85 10.3201/eid0901.020112 12533285 PMC2873753

[B13] Wang SM Liu CC Tseng HW Wang JR Huang CC Chen YJ Yang YJ Lin SJ Yeh TF Clinical spectrum of enterovirus 71 infection in children in southern Taiwan, with an emphasis on neurological complications Clin Infect Dis 1999 29 1 184 190 10.1086/520149 10433583

[B14] Zhang Y Tan XJ Wang HY Yan DM Zhu SL Wang DY Ji F Wang XJ Gao YJ Chen L An HQ Li DX Wang SW Xu AQ Wang ZJ Xu WB An outbreak of hand, foot, and mouth disease associated with subgenotype C4 of human enterovirus 71 in Shandong, China J Clin Virol 2009 44 4 262 267 10.1016/j.jcv.2009.02.002 19269888

[B15] Han J Ma XJ Wan JF Liu YH Han YL Chen C Tian C Gao C Wang M Dong XP Long persistence of EV71 specific uncleotides in respiratory and feces samples of the patients with Hand-foot-mouth disease after recovery BMC infectious disease 2010 10 178 10.1186/1471-2334-10-178 PMC2895604 20565813

[B16] Vatev N Stoycheva M Petrov A Venchev C Troyancheva M Prolonged viral excretion in faeces among patients with hepatitis A Euro Surveill 2011 16 19 137 14021748866

[B17] Richardson S Grimwood K Gorrell R Palombo E Barnes G Bishop R Extended excretion of rotavirus after severe diarrhoea in young children Lancet 1998 351 9119 1844 1848 10.1016/S0140-6736(97)11257-0 9652668

[B18] Yotsuyanagi H Koike K Yasuda K Moriya K Shintani Y Fujie H Kurokawa K Iino S Prolonged fecal excretion of hepatitis A virus in adult patients with hepatitis A as determined by polymerase chain reaction Hepatology 1996 24 1 10 13 10.1002/hep.510240103 8707246

[B19] Chen LJ Li RQ Zhang HR Wang YM He X Tracking investigating of the carriage status of virus among HFMD patients with EV71 and their close contacts Int J Virol 2011 18 5 134 138

[B20] Chung PW Huang YC Chang LY Lin TY Ning HC Duration of enterivirus shedding in stool J Microbiol Immunol Infect 2001 34 3 167 170 11605806

[B21] World Health Organization Polio laboratory manual 2004 4th Switzerland: Geneva

